# Verification of accuracy of qPCR method for intravital diagnostics of *Macrorhabdus ornithogaster* in avian droppings

**DOI:** 10.17221/85/2022-VETMED

**Published:** 2023-02-22

**Authors:** Lenka Vrbasova, Dobromila Molinkova, Petr Linhart, Zdenek Knotek

**Affiliations:** ^1^Avian and Exotic Animal Clinic, Faculty of Veterinary Medicine, University of Veterinary Sciences, Brno, Czech Republic; ^2^Department of Infectious Diseases and Microbiology, Faculty of Veterinary Medicine, University of Veterinary Sciences Brno, Brno, Czech Republic; ^3^Department of Animal Protection and Welfare and Veterinary Public Health, Faculty of Veterinary Hygiene and Ecology, University of Veterinary Sciences Brno, Brno, Czech Republic

**Keywords:** cytology, macrorhabdosis, passerines, psittacines

## Abstract

The objective of this study was to verify the accuracy of the quantitative PCR (qPCR) method for *in vivo* detection of *Macrorhabdus ornithogaster*. A total of 100 faecal and gastric mucosa samples from avian cadavers were investigated, using cytological and qPCR techniques (budgerigars, Fischer’s lovebirds, red-crowned parakeets, scarlet-chested parrots, eastern rosellas, domestic canaries, zebra finches, white Java sparrow). Using qPCR, the probability of detecting positive samples of droppings was significantly higher than in the faecal smear microscopy (*P* < 0.01). Cytology detected the presence of *Macrorhabdus ornithogaster* in 34 faecal samples, whereas qPCR detected 54 positive samples. In all 46 qPCR negative faecal samples, gastric smear qPCR was performed and also yielded negative results. Gastric smear qPCR was also performed in 20 cadavers where faecal qPCR has detected the presence of *Macrorhabdus ornithogaster* and in all samples confirmed the positive result. This verifies the accuracy of faecal sample qPCR for intravital diagnostics. Overall, the faecal qPCR technique appears to be extremely reliable, as it made it possible to detect all infected individuals, including those with negative stool or gastric cytology.

Macrorhabdosis is a worldwide widespread contagious avian disease of yeast origin (Tomaszewski et al. 2003). The aetiological agent of the disease, the anamorphic ascomycetous yeast *Macrorhabdus ornithogaster,* is described as a Gram-positive rod with rounded ends 20–90 μm long, 2–5 μm wide, and Y-shaped in exceptional cases. The organism’s ability to move has been described (Martins et al. 2006). It colonises the avian proventriculus and ventriculus, mainly in the isthmus (Phalen 2014). It has also not been identified in other organs, in the external environment or in mammals (Hanafusa et al. 2013). In many cases, it simply attaches to the mucous membrane without any signs of inflammation (Gerlach 2001; Hanafusa et al. 2007; Kheirandish and Salehi 2011). However, it can induce inflammatory changes in the gastric mucosa, increased mucus production, bleeding erosions, hyperplastic response of the mucosa. It adversely affects digestion in infected birds (Phalen 2014). Budgerigars with macrorhabdosis are significantly more likely to develop proventricular adenocarcinoma (Powers et al. 2019). The aetiological agent could be confirmed by post-mortem examination of gastric mucosal scrapings, impression cytology specimens or histologic examination of the stomach (Kheirandish and Salehi 2011; Phalen 2014). Hanka et al. (2010) reported only a single false negative result of histologic examination of the stomach in their study. *In* *vivo* diagnostics relies on cytology of native or stained droppings samples. However, it is problematic due to the intermittent faecal shedding of the aetiological agent and due to the existence of inapparent infections. Negative cytology results do not rule out infection as such. To some extent, the observed difference in the detection rates of *Macrorhabdus ornithogaster* by cytology may be influenced by the respective species and the patient’s clinical condition, sex, or type of aviary (Filippich and Hendrikz 1998; Vrbasova et al. 2020). Sample culture is not used for routine diagnostics due to the difficulty of the method (Hanafusa et al. 2007). Methods of agent concentration by homogenisation in saline solution, flotation technique (mini FLOTAC) and PCR have been described (Phalen 2014; Borrelli et al. 2015; Poleschinski et al. 2019; Baron et al. 2020). PCR of the yeast DNA in droppings appears to be the only sensitive and reliable intravital technique. The detection of positive individuals using this technique is significantly more reliable (Sullivan et al. 2017; Vrbasova et al. 2020) than in faecal cytology. The objective of this study was to determine the degree of accuracy of the faecal quantitative PCR (qPCR) technique for detecting *Macrorhabdus ornithogaster* in captive birds in the Czech Republic.

## MATERIAL AND METHODS

A total of 100 cadavers from 11 aviaries were examined (6 avian collections were positive for *Mac-rorhabdus ornithogaster*; the condition of 5 avian collections in terms of epizootiology was unknown). The group under investigation consisted of 8 bird species (Table 1). The orders *Psittaciformes* and *Passeriformes* were represented by 83 individuals belonging to 5 species and 17 individuals from 3 species, respectively (Table 2). Sex distribution across the population was 46 males and 54 females. All the cadavers were subjected to faecal cytology, gastric smear cytology and faecal qPCR (Table 3).

**Table 1:** Characteristics of aviaries and birds examined for the presence of *Macrorhabdus ornithogaster*

Characteristics	Number
Number of bird cadavers examined	100
Number of avian collections examined	11
Number of avian collections examined with *Macrorhabdus ornithogaster-positive* birds in the previous study	6
Number of birds examined from avian collections with *Macrorhabdus ornithogaster* positive birds in the previous study	95
Number of aviaries with unknown history of *Macrorhabdus ornithogaster*	5
Number of birds from aviaries with unknown history of *Macrorhabdus ornithogaster*	5
Number of psittacine birds examined	83
Number of passerine birds examined	17
Number of males examined	46
Number of females examined	54

**Table 2:** Birds’ species examined for the presence of *Macrorhabdus ornithogaster*

Species		Total number	Males	Females
Budgerigars	*Melopsittacus undulatus*	66	30	36
Fischer’s lovebird	*Agapornis fischeri*	13	8	5
Red-crowned parakeet	*Cyanoramphus novaezelandiae*	2	2	–
Scarlet-chested parrot	*Neophema splendida*	1	–	1
Eastern rossella	*Platycercus eximius*	1	1	–
Domestic canary	*Serinus canaria domestica*	4	–	4
Zebra finches	*Taeniopygia guttata*	12	4	8
White Java sparrow	*Padda oryzivora*	1	1	–

**Table 3:** Results of diagnostics *Macrorhabdus ornithogaster* with the use of different diagnostic techniques

Category	Number (*n*)	Techniques used for the determination of the *Macrorhabdus ornithogaster*							
		cytology of the faecal samples			qPCR of the faecal samples			cytology of the gastric mucosal smears	
		positive	negative		positive	negative		positive	negative
All cadavers	100	34 34%	66 66%		54 54%	46 46%		50 50%	50 50%
Males	46	13 28.26%	33 71.74%		21 45.65%	25 54.35%		18 39.13%	28 60.87%
Females	54	21 38.89%	33 61.11%		33 61.11%	21 38.89%		32 59.26%	22 40.74%
Psittacines	83	24 28.92%	59 71.08%		40 48.19%	43 51.81%		36 43.37%	47 56.63%
Passerines	17	10 58.82%	7 41.18%		14 82.35%	3 17.65%		14 82.35%	3 17.65%

The autopsy, sampling, and cytology were performed in the autopsy room and in the outpatient clinic of the Avian, and Exotic Animal Clinic of the University of Veterinary Sciences Brno. A faecal smear and an impression specimen of the gastric mucosa were made for each individual. After drying, the samples were heat-fixed, Diff-Quik stained, and thoroughly investigated by the same person using an optical microscope with immersion (× 1 000 magnification). A sample of droppings and gastric smears for qPCR were also collected from each cadaver. Droppings samples and gastric smears for qPCR were placed individually in resealable bags and stored at –18 °C. DNA isolation and purification for qPCR and the qPCR assays were performed in the laboratories of the Department of Infectious Diseases and Microbiology of the University of Veterinary Sciences Brno.

For faecal qPCR, 180 mg of the sample was used for DNA extraction. NucleoSpin DNA kit (Macherey-Nagel, Düren, Germany) was used for DNA isolation and purification in line with the manufacturer’s instructions. The extracted DNA solution was stored at –18 °C. Negative control was included in each set to check for possible contamination (false positives) at all times. The NucleoSpin DNA blood kit (Macherey-Nagel, Düren, Germany) was used for DNA extraction from gastric mucosal smears. The gastric smears were placed in collection tubes with 500 μl of phosphate-buffered saline (PBS), vortex mixed for 5 s, then the manufacturer’s instructions were followed. The extracted DNA was stored at –18 °C. Negative control was included in each set to check for possible contamination at all times.

Composition of the reaction mixture per sample: 10 μl of Luna^®^ Universal qPCR Master Mix (New England BioLabs, Ipswich, USA); 6 μl of PCR H_2_O; 400 nM of forward primer (sequence 5'-3'GGGATCGGGTGGAGTTTAAATAG); 400 nM of reverse primer TTTCAGCCTTGCGACCATAC (product: position 1 016–1 110; 94 bp); 2 μl of template DNA. The reaction was carried out in the Light-Cycler 480 Real-Time PCR Instrument (Roche, Basel, Switzerland) according to the following program: initial denaturation at 95 °C for 3 min, 40 cycles: denaturation at 95 °C for 5 s, annealing and extension at 60 °C for 30 s terminated by cooling at 40 °C for 30 minutes). Negative and positive controls were used for each assay. For all samples, the amplification curve was read, and the denaturation midpoint was verified by comparing the melting curve with the positive control.

To verify the accuracy of faecal qPCR for intravital diagnostics of *Macrorhabdus ornithogaster*-positive birds, qPCR of the gastric smear was performed on all negative samples (46 samples) after reading the faecal qPCR. Accuracy was also verified by subjecting 20 positive cadavers (as determined by faecal qPCR) to paired qPCR of the gastric smear (Table 4).

**Table 4:** Verification of accuracy of faecal qPCR

qPCR of thefaecal samples		Cytology of thefaecal samples		qPCR of the gastricmucosal smears		Cytology of the gastricmucosal smears	
Category	(n)	positive	negative	positive	negative	positive	negative
All negative samples	46	0	46 100%	0	46 100%	0	46 100%
Selected positive samples	20	11 55%	9 45%	20 100%	0	16 80%	4 20%

The frequency of positive detections of *Mac-rorhabdus ornithogaster* was analysed by Chi-square test using contingency tables. All analyses were performed using the Unistat statistical software (v6.0.07; UNISTAT Ltd., UK).

## RESULTS

Of the 100 droppings samples investigated, faecal cytology detected *Macrorhabdus ornithogaster* in 34 samples only, whereas faecal qPCR detected 54 positive stool samples (Table 3). The probability of detecting the aetiological agent was therefore statistically significantly higher using the qPCR technique than the faecal smear cytology (*P* < 0.01). In 34 out of the 54 qPCR-*Macrorhabdus ornithogaster*-positive individuals (62.96%), the shedding of *Macrorhabdus ornithogaster* was also confirmed by faecal cytology. Cytology of the stomach wall impression detected 50 positive samples, which were not significantly different from the results obtained by faecal qPCR (54 positive samples) (*P* > 0.05).

In order to verify the accuracy of the faecal qPCR method for intravital diagnostics of *Macrorhabdus ornithogaster*, all 46 avian cadavers with prior negative results of faecal qPCR were also subjected to subsequent qPCR of the gastric smear. The gastric smear qPCR also yielded negative results in all the negative faecal qPCR samples (100%). Gastric smear qPCR was also performed in 20 cadavers where faecal qPCR had detected positivity for *Macrorhabdus ornithogaster*. All 20 samples were also positive when using qPCR of the gastric smears. The faecal qPCR method made it possible to detect all infected individuals, including those with negative droppings or negative gastric cytology (Table 4).

Using the qPCR technique to detect the presence of *Macrorhabdus ornithogaster*, 21 out of a total of 46 male birds were positive (45.65%) and 33 out of a total of 54 female birds were positive (61.11%) (Table 3), but no significant difference was found between the positive findings and the sex of the cadavers examined (*P* = 0.178 8). The 61.90% of the qPCR-positive males (13/21) and 63.63% (21/33) of the qPCR-positive females shed a cytologically detectable aetiological agent in their droppings. The sex difference in faecal shedding of the cytologically detectable pathogen was not significant (*P* > 0.05).

The qPCR method used to detect the presence of *Macrorhabdus ornithogaster* identified 48.19% of positive psittacines (40/83) and 82.35% of passerines (14/17) (Table 3). Using this technique, the prevalence observed was significantly higher in passerines than in psittacines (*P *< 0.05). The incidence rates of *Macrorhabdus ornithogaster* were significantly higher (*P* < 0.05) in passerines (58.82%) than in psittacines (28.91%) in faecal cytology as well.

## DISCUSSION

The *Macrorhabdus ornithogaster* infection poses a serious health risk to both captive and free-living birds (Marlier et al. 2006; Piasecki et al. 2012). To prevent the spread of infection accurate diagnostics of positive individuals is important. Faecal quantitative PCR is considered to be a highly sensitive technique for intravital diagnostics of *Macrorhabdus ornithogaster* in birds. The difference between faecal cytology and PCR in the success rates of detecting positive individuals varies considerably in the literature. Differences among species as well as among the aviaries under investigation were also recorded (Sullivan et al. 2017; Pustow and Krautwald-Junghanns 2017; Vrbasova et al. 2020). Phalen (2014) recommends collecting 5 faecal samples to increase the detection rate of positive individuals using faecal cytology. In 2003, Phalen and Moore described a tenfold multiplication of the aetiological agent 14 days after inoculation of 1-day-old chicks (Phalen and Moore 2003). Increased probability of cytological detection of the aetiological agent in droppings collected 14 to 21 days after purchase can be thus expected in newly acquired birds, for whom the change of environment is a stress factor. The results of our study suggest that the difference between the detection rates of *Macrorhabdus ornithogaster* in faeces by cytology and by qPCR was highly significant (*P* < 0.01), and that faecal qPCR can detect positive individuals, with up to 100% accuracy in suspected cases, which greatly favours qPCR compared to other techniques.

Comparing the results of faecal cytology and gastric impression cytology with faecal qPCR, the latter appears to be a highly reliable method for intravital testing of captive birds. All the samples were consistent with the qPCR results of the paired gastric smear samples.

Compared to faecal qPCR, the cytology of the gastric impression is relatively reliable and constitutes a suitable alternative if an autopsy is performed. Hanka et al. (2010) have reported a false negative result in cytology of the gastric mucosal impression in birds only occasionally. In our cohort, we had 4 false negatives out of the 54 positive samples. Having simultaneously assessed gastric histology and gastric impression cytology, Kheirandish and Salehi (2011) reported no false negatives. Histologic examination of tissues from budgerigars infected with *Macrorhabdus orthithogaster* identified an apparent continuum in the development of proventricular isthmus lesions associated with *Macrorhabdus ornithogaster* that included inflammation, mucosal hyperplasia, glandular dysplasia, and adenocarcinoma (Powers et al. 2019).

Using faecal cytology for budgerigars in two colonies, Filippich and Hendrikz (1998) reported a significantly higher prevalence of *Macrorhabdus ornithogaster* in males than in females (*P* < 0.05) and also a significant difference in prevalence among the avian collections under investigation. It has not been confirmed that detection rates of *Macrorhabdus ornithogaster* in the investigated droppings depend on the budgerigars’ age (Filippich and Hendrikz 1998). The cohort of positive individuals investigated by us included higher numbers of females than males, but no significant influence of sex has been confirmed. The age of the birds under investigation was not compared in our study. We did not compare how the quality of each avian collection affected the detection rates of *Macrorhabdus ornithogaster*. More research is needed for further progress on this topic.

The prevalence of *Macrorhabdus ornithogaster* is highly variable and can range from 22.5% to 64% in budgerigars and from 9% to 55% in canaries (Lanzarot et al. 2013). Piasecki et al. (2012) confirmed *Macrorhabdus ornithogaster* in 65% of budgerigars and only in 9.3% of canaries. Although the numbers of psittacines and passerines investigated in our study were quite different (83 and 17, respectively), we observed a significant difference in the number of *Macrorhabdus ornithogaster*-positive individuals between the *Psittaciformes* and *Passeriformes* using both faecal cytology and qPCR*.* In contrast, Marlier et al. (2006) reported *Macrorhabdus ornithogaster* as a likely cause of death in 22.5% of budgerigars and 28% of canaries in their retrospective study.

In conclusion, it could be stated that irregular faecal shedding of *Macrorhabdus ornithogaster* may lead to overlooking infected birds when performing faecal cytology. The faecal qPCR technique made it possible to detect all infected individuals, including those with negative faeces or gastric cytology. The results of our study suggest that faecal qPCR is a reliable technique applicable in the intravital diagnostics of *Macrorhabdus ornithogaster*.
